# Modification of the Dielectric and Thermal Properties of Organic Frameworks Based on Nonterminal Epoxy Liquid Crystal with Silicon Dioxide and Titanium Dioxide

**DOI:** 10.3390/polym16101320

**Published:** 2024-05-08

**Authors:** Lidia Okrasa, Magdalena Włodarska, Maciej Kisiel, Beata Mossety-Leszczak

**Affiliations:** 1 Department of Molecular Physics, Lodz University of Technology, Żeromskiego 116, 90-924 Lodz, Poland; 2 Institute of Physics, Lodz University of Technology, Wólczańska 217/221, 93-005 Lodz, Poland; magdalena.wlodarska@p.lodz.pl; 3 Department of Industrial and Materials Chemistry, Rzeszow University of Technology, al. Powstańców Warszawy 12, 35-959 Rzeszow, Poland; m.kisiel@prz.edu.pl (M.K.); mossety@prz.edu.pl (B.M.-L.)

**Keywords:** composites, liquid crystal epoxy resins, dielectric spectroscopy, SiO_2_, TiO_2_

## Abstract

A nonterminal liquid crystal epoxy monomer is used to create an epoxy–amine network with a typical diamine 4,4′diaminodiphenylmethane. The plain matrix is compared to matrices modified with inorganic fillers: TiO_2_ or SiO_2_. Conditions of the curing reaction and glass transition temperatures in the cured products are determined through differential scanning calorimetry and broadband dielectric spectroscopy. The curing process is also followed through optical and electrical observations. The dielectric response of all investigated networks reveals a segmental α-process related to structural reorientation (connected to the glass transition). In all products, a similar process associated with molecular motions of polar groups also appears. The matrix modified with TiO_2_ exhibits two secondary relaxation processes (β and γ). Similar processes were observed in the pure monomer. An advantage of the network with the TiO_2_ filler is a shorter time or lower temperature required for optimal curing conditions. The physical properties of cured matrices depend on the presence of a nematic phase in the monomer and nonterminal functional groups in the aliphatic chains. In effect, such cured matrices can have more flexibility and internal order than classical resins. Additional modifiers used in this work shift the glass transition above room temperature and influence the fragility index in both cases.

## 1. Introduction

Modern materials are required to have advanced functionalities while retaining durability, mechanical, thermal and chemical resistance. Epoxy resins are well suited for many tasks due to their ease of production and great susceptibility to modification. To obtain a product with desired properties, it is crucial to adjust specific factors on which these properties depend. The selection of the epoxy precursor and the hardener is very important, as their molecular structure has a deep impact on the structure of the final network. Another factor is the curing temperature and the reaction time [1,2,3,4]. Epoxy matrices can also be used as a basis for advanced composites with added or modified properties [5,6]. An example of the development of epoxy materials is the use of liquid crystal (LC) epoxy precursors to produce liquid crystal epoxy networks (LCEN), which can be divided in two classes: liquid crystal thermosets and elastomers [7,8,9,10,11,12]. The first class is characterized by a frozen structure with a local or global LC-like orientation. In the second class, the orientation can be forced by thermal or mechanical factors [7,8,9,10,11,12]. Such composites may have favorable properties. For instance, it was shown that LCEN thermosets have very good effectiveness in heat dissipation (better than traditional epoxy resins) and can be successfully used in electronic circuits [13]. When cured with typical hardeners, LCENs tend to form rigid matrices [1,2,9,10,11]. A frequent problem with these materials is that they are often brittle, which is considered a disadvantage. Hence, there is continued research effort aimed at improving their quality for commercial applications by creating more elastic composites without simultaneously losing properties such as high chemical resistance or good thermal conductivity. An attempt to improve elasticity by adding plasticizers was not satisfactory because it decreased the durability of the final products and significantly changed their thermal properties. A different approach was to increase the elasticity of polymer networks by manipulating the molecular structure of the precursors [3,4]. In our work, we used a new nonterminal epoxy monomer (NTEM) as a precursor with a low glass transition temperature (*T_g_*), which was expected to improve the elasticity of the LCEN. However, the *T_g_* of the obtained network was too low, and too close to room temperature. To solve this problem, we decided to use popular fillers whose typical use to date tends to increase *T_g_* in composites as well as improve stiffness of the products. Such inorganic compounds are often used to stabilize organic frameworks [14]. For the present study, we chose silicon dioxide and titanium dioxide as fillers. Both are in broad use as components of various types of composites in many branches of the industry, particularly as fillers and reinforcing agents. Metal oxides are often used as modifiers in liquid crystals, polymer-dispersed liquid crystal composites and composites based on epoxy resins, including epoxies containing mesogens [15,16,17,18]. A broad description of the interaction of metal oxides with liquid crystals was presented in [15]. Within this extensive summary, one can also find the interaction with TiO_2_ or SiO_2_ and their influence on the dielectric and electro-optical properties, and the possibility of using such mixtures for polymer-dispersed liquid crystal composites [15]. In [16], it was shown that the addition of TiO_2_ significantly changes the properties of the mixture, and a detailed description of this interaction was given depending on the size of the modifier, while in [17], SiO_2_ was used to modify the electro-optical properties of polymer-dispersed liquid crystals. Metal oxides are also used to improve thermal and mechanical properties in epoxy composites, including epoxy compounds with mesogenic units [18,19,20]. Additionally, titanium dioxide is known for its photocatalytic activity and protective properties against the degradative effects of weather conditions, especially UV radiation [21,22,23]. The modification of pure networks based on NTEM is expected not only to improve their elastic properties and increase heat dissipation, but also to make them more stable by increasing *T_g_* above room temperature.

One means of analyzing the macroscopic properties of new materials and composites is broadband dielectric spectroscopy (BDS). This technique provides insight into the molecular motions contributing to the dielectric relaxation processes in the studied material, which can then be correlated with the polymer network’s macroscopic physical properties. The complex dielectric permittivity data can be visualized over a broad frequency range, covering many decades from very low to very high frequencies, and over a range of temperatures. In such a 3D view, changes in the molecular motions (reflected in the relaxation processes) can be easily observed [24,25,26]. Such motions can be related to macroscopic physical properties, and they are dependent on both the overall microscopic structure of the studied material and the presence of polar groups [24]. In particular, in a wide variety of polymers tested experimentally, the segmental α-process was found to be correlated with structural changes [24,25,26]. The vanishing of the α-mode during cooling allows establishing the glass transition temperature. It also enables the determination of some parameters which gives grounds to reasoning about the fragility of the material. The standard interpretation of β- and γ-processes associates their origins with the mobility of polar groups or small parts of the molecule. Therefore, the actual molecular structure must be considered when drawing a detailed interpretation [24,25,26]. For example, the γ-process is usually correlated with fluctuations of the carbon chain conformation, while the β-process can be associated with the reorientational movements of the polar groups attached as side groups to the main chain in the polymers, or to the polymer network [24].

With BDS one can obtain several parameters connected with the physical properties of the investigated materials. This is commonly performed by approximating experimental data with the following semi-empirical equation, which can successfully describe most relaxation processes observed in a broad range of materials, originally proposed by Havriliak and Negami [27], with an added DC conductivity contribution [24,28]:

(1) $$\varepsilon^{*}(\omega )-\varepsilon_{\infty }=-i\frac{\sigma_{0}}{\varepsilon_{0}\omega^{s}}+\sum_{k}\frac{\Delta \varepsilon_{k}}{(1+(i\omega \tau_{k}{)}^{\alpha_{k}}{)}^{\beta_{k}}}$$

The observed relaxation process can be conveniently studied by looking at the values of the characteristic parameters controlling the shape of the HN function: *τ_k_* (relaxation time), *α_k_* and *β_k_* (shape parameters) and *σ*_0_ (DC conductivity). In this expression, the electric permittivity of vacuum is symbolized by *ε_o_*; *ε*_∞_ is the extrapolated value of *ε*′ at infinite frequency; and *ω* and Δ*ε* are, respectively, the radial frequency and the dielectric strength. Non-ohmic effects are accounted for in the *s* parameter. The value calculated from the HN equation stems from its ability to describe different relaxation processes with the same set of characteristic parameters, which can then be compared across different materials. The conclusions drawn here allow one to connect individual relaxation processes to properties of the studied materials. Therefore, HN formalism is also a good basis for comparing these properties.

The α-process originates from structural changes in the material and is commonly associated with segmental mobility in the polymer chain. In particular, glass transition is correlated with the vanishing of this process. The empirical Vogel–Fulcher–Tammann (VFT) formula is a good approximation of the dependency of the relaxation time τ of this process on temperature *T* [25]:

(2) $$\ln(\frac{1}{\tau })=\frac{-DT_{V}}{T-T_{V}}+\ln(\frac{1}{\tau_{0}}),$$

where *τ*_0_ is the relaxation time in the limit of high temperatures, *D* is the fragility index describing a deviation of temperature dependence of relaxation time *τ* from the Arrhenius law and *T_V_* is the so-called Vogel temperature, which sometimes is identified as the “ideal” glass transition temperature [29]. From the VFT relationship, one can determine the glass transition temperature (the temperature at which the relaxation time of the α relaxation process reaches 100 s) as well as other parameters. One of them is the dynamic fragility index *m*, which can be determined from the following formula:

(3) $$m=\frac{\;DT_{V}}{2.3\cdot T_{g}(1-\frac{T_{V}}{T_{g}}{)}^{2}}$$

The “fragile” liquids (low *m* values) exhibit more rapid changes of relaxation times than the “strong” liquids (high *m* values) in the temperature range rising through the glass transition region.

Another parameter that can be calculated is the apparent activation energy *E_a_*′ at *T_g_* using the following formula [30]:

(4) $$E_{a}^{\prime }\left(T_{g}\right)=\frac{\;RDT_{V}}{(1-\frac{T_{V}}{T_{g}}{)}^{2}},$$

where *R* is the universal gas constant and is equal 8.314 J mol^−1^ K^−1^.

On the other hand, relaxation times of secondary processes related to local motions (β- and γ-processes) follow the Arrhenius dependency:

(5) $$\ln(\frac{1}{\tau })=\frac{-E_{a}}{kT}+\ln(\frac{1}{\tau_{0}})$$

with the characteristic activation energy *E_a_*, which can also be compared across different processes and materials.

In addition to studying the properties of the obtained networks, electric measurements are a convenient tool for monitoring of curing reactions in epoxy resins [31,32,33,34]. The advancement of the curing reaction is reflected in observable transformations occurring in dielectric relaxation processes [31,32,33]. It can also be monitored in situ by following changes in electric conductivity [34]. For materials crosslinked at low temperatures, dielectric spectroscopy is used to observe changes in conductivity, while at elevated temperatures direct conductivity measurements in a constant electric field can be used. In our experiments, the progress of the reaction was monitored through optical observations as well as through constant monitoring of the electric conductivity during curing.

In this paper, two modifications of an epoxy thermoset based on a nonterminal epoxy nematic monomer are compared. We attempt to increase the stabilization and vitrification temperature and to modify the fragility of the final network by introducing titanium dioxide or silicon dioxide to the reaction mixture. We focus on studying the conditions of the curing process, changes in the glass transition and molecular relaxations in all the investigated matrices. The observed differences suggest that the matrix with fillers preserves all the advantages of the plain epoxy network while shifting the glass transition above the room temperature, which makes the final material more stable at room conditions.

## 2. Materials and Methods

### 2.1. Materials

The epoxy networks obtained in this study were based on a newly synthesized liquid crystal epoxy compound (NTEM) with three aromatic rings in the mesogen and symmetrically attached long aliphatic chains with nonterminal functional groups. Phase transitions in the investigated monomer are presented in Table 1. The reader can refer to an earlier paper [35] for the full details of the synthesis of NTEM. That report also described phase transitions and presented a preliminary investigation of the curing conditions [35].

**Scheme 1:**
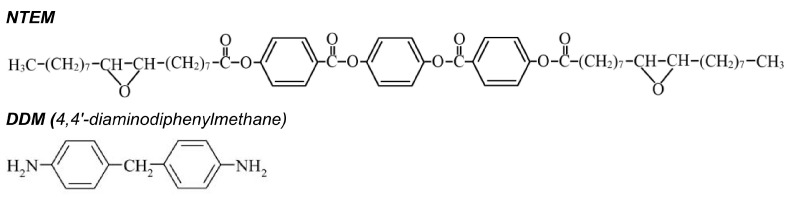
Molecular structure of the matrix precursors: the epoxy monomer and the diamine.

The epoxy monomer was cured with a typical amine, 4,4′-diaminodiphenylmethane (DDM). The molecular structures of the monomer and the curing agent are shown in Scheme 1.

The mechanism of the epoxy crosslinking reaction depends on the choice of the curing agent [34,36]. The hardener and the crosslinking conditions have a significant impact on the physical properties of the obtained product [34,36]. Aromatic amines (including DDM) are very popular hardeners for epoxy materials, and they are usually used in a stoichiometric ratio which yields stiff and stable matrices [34,35]. The curing conditions required to obtain a fully cured matrix based on the investigated monomer were discussed in paper [35] and compared with another monomer and the same DDM amine. In the present work, for a better comparison of the physical properties, modified matrices were prepared with the addition of TiO_2_ or SiO_2_ induced into the mixture before the curing process. As described in the Introduction, the typical amounts of oxide additives used in liquid crystal and polymer-dispersed liquid crystal composites are only 2–3%, because they have a very large impact on the dielectric and electro-optical properties in the liquid crystal state [16,17]. However, in the case of epoxy resins, a higher content of the additive is common due to their smaller impact on the properties of solids [18]. Therefore, in our work we used concentrations of about 10% to observe changes in the products obtained after crosslinking. Compositions of the individual mixtures are collected in Table 2 along with the assigned acronyms. All reagents were obtained from commercial sources, including Warchem, Chempur, Sigma-Aldrich, POCH-Gliwice, Fisher Chemicals and Fluka. No additional purification was applied to the purchased chemicals.

### 2.2. Experimental Technique

The real and imaginary components of the complex dielectric permittivity of all the cured polymer matrices were measured using a broadband, high-resolution dielectric analyzer (Novocontrol Alpha) combined with a cryosystem (Quatro)- both manufactured by NOVOCONTROL Technologies GmbH & Co. KG, Montabaur, Germany- controlled by WinDETA software version 5.91. The temperature during the measurements was stabilized with the accuracy of 0.1 K. The error in determining the dielectric permittivity is so small that it does not significantly affect the results. An example of a plot of the imaginary part of the complex dielectric permittivity with error bars is shown in Figure S1 in the Supplementary Data. Pulverized mixtures were placed between 10 mm diameter parallel metal electrodes, separated with 0.02 mm Teflon spacers. The precision of the equipment allows us to obtain relative values of the complex dielectric permittivity. However, in situ crosslinking of the mixture between the electrodes makes it difficult to determine accurately the final geometry of the obtained samples (some pores may occur in it). The analysis of dielectric spectra (deconvolution of the spectrum into individual relaxations and determination of their relaxation times) was carried out using the WinFit software version 3.5, which uses the previously described HN equation with the added DC conductivity contribution (Equation (1)). An example of deconvolution of selected dielectric spectra is shown in Figures S2 and S3 in the Supplementary Data. The obtained characteristic relaxation times, independent of the sample geometry, were further used to determine the parameters of the VFT formula.

The process of curing was also conducted isothermally, with a temperature stabilization accuracy of 0.5 K provided by a temperature controller (UNIPAN 660, Zakład Aparatury Naukowej “UNIPAN”, Warsaw, Poland). The experimental setup used for electrical observations of this process consisted of a programmable digital switching mode DC regulated power supply (Manson SDP 2803, Manson Engineering Industrial Ltd., Kwai Chung N.T., Hong Kong) and a digital multimeter (Fluke 8808A, Fluke Corp., Everett, WA, USA), controlled by specialized computer software developed internally in the Technical University of Lodz.

A Calorimeter DSC3+ (Mettler-Toledo GmbH, Greifensee, Switzerland) equipped with dedicated STARe software (version 16.10) was used in calorimetric studies of both the final products and the crosslinking process. Thermograms were recorded in the heating and cooling routes at a constant rate of 10 K min^−1^, under nitrogen flow. The calorimeter was calibrated using In and Zn standards (Mettler Toledo).

Optical observations of the crosslinking process of the mixtures were made by depositing the powder directly on glass in a hot stage (Linkam HFS600E-PB4, Linkam Scientific Instruments Ltd., Redhill, UK). Microscopic images of the subject in a crossed-polarizer arrangement were captured using a digital camera (Moticam 2.0, Motic Europe S.L.U., Barcelona, Spain).

## 3. Results and Discussion

### 3.1. Curing Conditions

**Figure 1:**
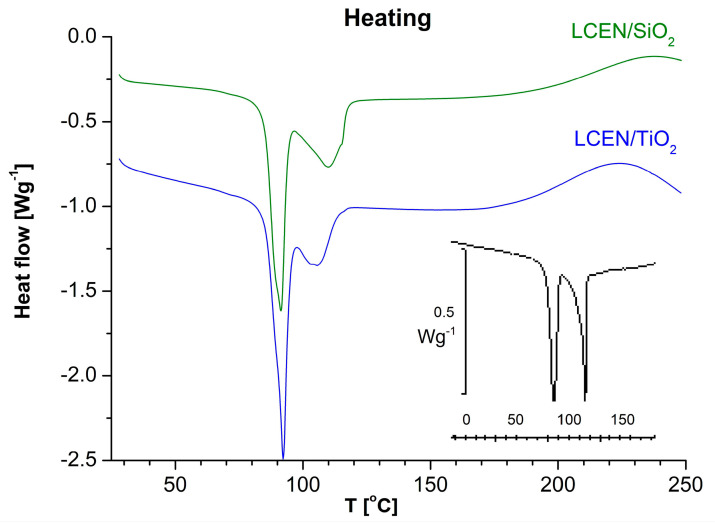
DSC thermograms of the curing process (heating route) for mixtures based on the investigated monomer cured with DDM and with SiO_2_ or TiO_2_ fillers. Inset: thermograms for the pure mixture of the monomer with DDM [35].

Figure 1 presents the differential scanning calorimetry (DSC) thermogram of the LCEN/SiO_2_ mixture (heating route) in addition to the corresponding thermogram acquired for the LCEN/TiO_2_ composition.

Both thermograms reveal similar melting points of individual components of the mixtures—below and above 100 °C. Due to the crosslinking that occurs during this heating, these two processes disappeared in crosslinked materials. (It is well seen in Figure S4 in the Supplementary Data and in DSC thermograms of the cured epoxy matrices discussed later). Adjusting the crosslinking conditions, such as the temperature and crosslinking time, is a very important step that influences the physical properties of the obtained product. Additionally, each mixture behaves slightly differently depending on its composition, and therefore the crosslinking temperature and time are adjusted separately for specific mixtures. Firstly, the reaction with amine DDM takes place at an elevated temperature, with a characteristic heat flow increase and an exothermic peak on DSC thermograms, which indicates the temperature range at which the reaction occurs. As the temperature increases, the reaction rate increases as well. In our case, the curing reaction produces an exothermic peak which appears clearly in the temperature range located slightly over 180 °C in LCEN/TiO_2_. The same peak is less prominent in LCEN/SiO_2_, where the heat flow starts increasing at temperatures above 200 °C. Based on these observations, temperatures for isothermal crosslinking can be selected, and it is apparent that LCEN/TiO_2_ needs a slightly lower curing temperature (or shorter time) than its counterpart. This is advantageous because lower curing temperatures stimulate a smoother course of the reaction, at the expense of a longer crosslinking time. Lower curing temperatures are desirable even if extra annealing is sometimes needed to obtain a stable final product. The curing process of pure mixtures presented in the earlier work [35] took place at a similar temperature, but the heat flow increase and the curing peak were less visible. With DSC, one can identify the crosslinking temperatures, but the reaction time depends on the selected temperature (at higher temperatures, the reaction runs faster), which requires additional observations to determine that time. Table 3 presents the actual temperatures selected for the two investigated systems, along with the reaction time selected on grounds of the optical and electrical observations described later in this work. The obtained values are compared with the curing conditions used with the pure epoxy mixture [35].

**Figure 2:**
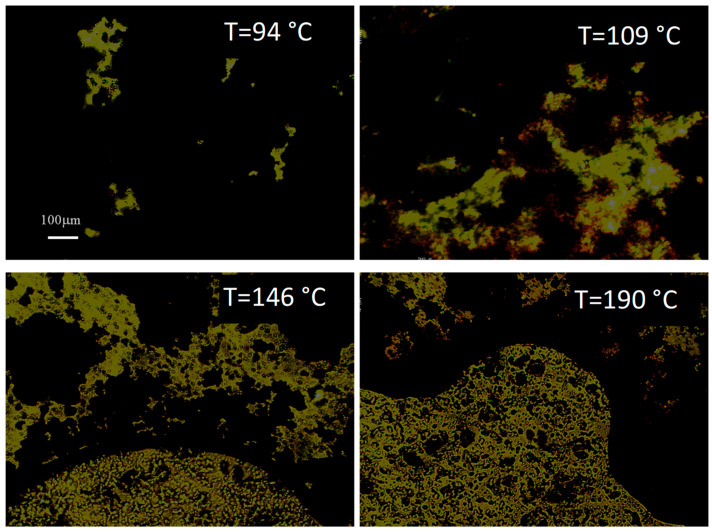
Optical observations of the epoxy–amine mixture with TiO_2_ filler during heating. Microscopic images between crossed polarizers acquired at a few selected temperatures.

For confirmation of the appearing phase transitions and the crosslinking process, observations in a polarizing microscope were made. During heating and in the microscopic observations, phase transitions (melting of components) can be observed through changes in the texture. After the material is heated to the crosslinking temperature, further observations allow us to track changes in the texture during the reaction. The observed changes illustrate the matrix formation process. After some time, no further changes are visible as the mixture reaches a high conversion ratio. An example of microphotographs captured for the LCEN/TiO_2_ mixture is shown in Figure 2, where changes in phase transitions during heating and after curing are presented.

Because of the liquid crystal nature of the precursor, observations can be performed in polarized light, in the arrangement with crossed polarizers. The obtained polymer matrix has anisotropic properties, so in polarized light it is also possible to monitor changes in the optical image during isothermal curing. The time elapsed until no further changes occur within the view can be taken as the lower estimate of the time required for completion of the curing reaction. Also, further observations of the hardening products both during cooling and reheating did not change the image in the polarizing microscope. Durable and solid hardened materials could be used for further measurements.

**Figure 3:**
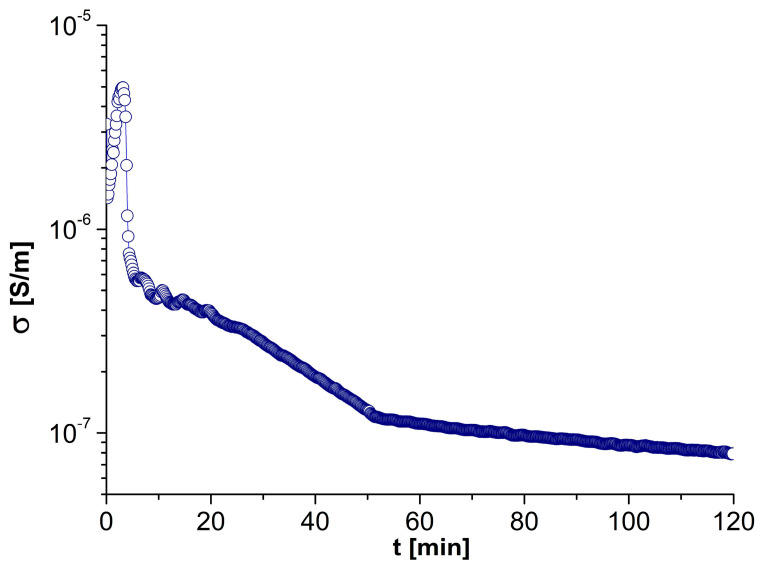
An example of the observation of changes in the electric conductivity with time during the isothermal curing of an epoxy matrix with TiO_2_ filler at 190 °C.

The progress of the curing process can also be tracked with electric measurements. Measurements carried out in situ facilitate a continuous assessment of the degree of conversion. They can also help determine the overall duration of the curing reaction at a given temperature. A representative example of the electric response of the LCEN/TiO_2_ mixture during isothermal curing at 190 °C is shown in Figure 3. The significant changes in the DC conductivity observed in Figure 3 during the first few minutes illustrate the reaction onset, when the material in the liquid state is just starting to solidify. This rapid exothermic reaction stage is followed by a slower phase in which the structure of the material stabilizes, which is visible in Figure 3 as a slow decrease in the conductivity. Similar observations were also carried out in other epoxy systems with different hardeners, which also allowed us to observe different reaction kinetics depending on the selected hardener [34].

Gradual changes in the electric conductivity prove that curing continuously progresses for about one hour in that case; after that time, further changes in the conductivity are minimal but they still allow us to follow the curing progress. Similar observations were also made for the other matrix. In summary, both the optical and electric observations helped determine the optimal reaction time for isothermal curing at the chosen temperature.

**Figure 4:**
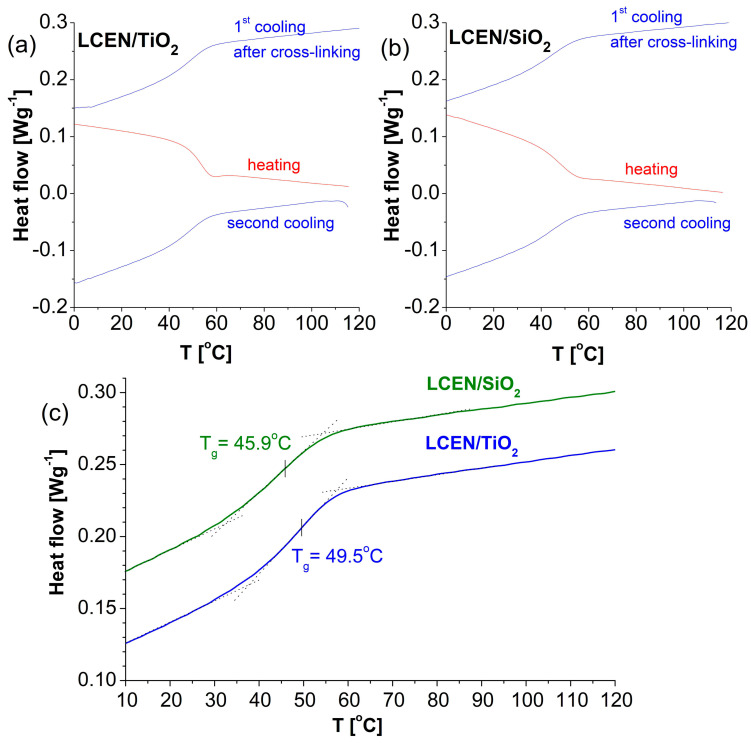
DSC thermograms of the cured epoxy matrices with SiO_2_ (**a**) and TiO_2_ (**b**) fillers. The graph (**c**) shows the determination of *T_g_* for the first cooling.

Based on all observations, optimal curing conditions were determined and summarized in Table 3. These conditions were then used to crosslink materials for further studies. The created matrices no longer displayed phase transitions during the heating and cooling cycle; only the glass transition appeared above room temperature. Figure 4 presents this transition for both modified matrices. The values of the glass transition temperature obtained from the DSC measurements during the first cooling after crosslinking according to the procedure presented in Table 3 (Figure 4c) were compared with other parameters and presented in Table 4 at the end of this work. After the crosslinking presented in Figure 3 and cooling (described above), the second heating up to 120 °C and cooling was performed. All three runs (cooling, heating and second cooling) are shown in Figure 4. The results of these measurements confirmed the stability of the obtained crosslinked materials. The DSC thermogram for the pure matrix was shown in manuscript [35].

### 3.2. Dielectric Observation of the Examined Materials

**Figure 5:**
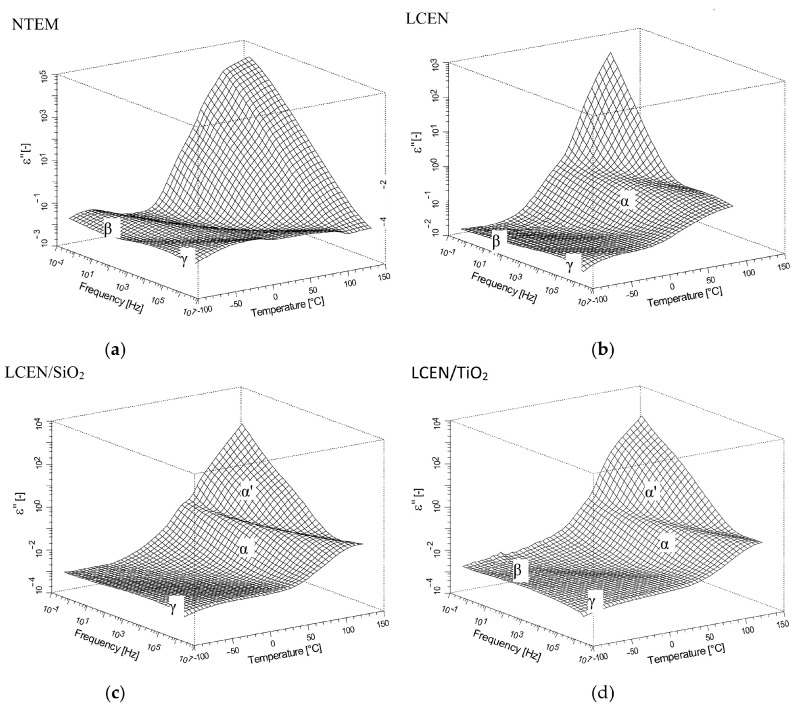
Dielectric relaxations in all the examined materials in 3D representation of the imaginary part of the complex dielectric permittivity vs. frequency and temperature with characteristic β- and γ-processes at low temperatures; (**a**) monomer, (**b**) plain epoxy network, (**c**) epoxy network with SiO_2_, and (**d**) epoxy network with TiO_2_.

Measurements of the imaginary component (*ε*″) of the complex dielectric permittivity of the investigated mixtures were performed in a broad range of frequencies and temperatures. The values of *ε*″ are plotted in Figure 5 along with the analogous results for the pure epoxy matrix (LCEN) and the epoxy precursor (NTEM). In this representation, characteristic relaxation processes appear very clearly, and they can be linked with the properties of the materials. The dielectric response of the monomer is generally different from the other three at elevated temperatures, though two relaxations processes (β, γ) appearing at low temperatures are similar in the monomer and in the plain matrix (Figure 5a,b). In the next phase of the study, we acquired and compared the dielectric response of both cured matrices. Two relaxation processes (α, γ) appear in all the cases, although the DC conductivity partially covers the α-process at high temperatures. Below the glass transition temperature, DC conductivity decreases quite significantly, which is visible in all matrices (Figure 5b–d). An additional β-process is observed at low temperatures in the plain matrix (LCEN) and in the matrix with the TiO_2_ modifier (LCEN/TiO_2_)—Figure 5b,d. In both matrices with modifiers, a relaxation called the α′-process is also noticeable at high temperatures (Figure 5c,d).

**Figure 6:**
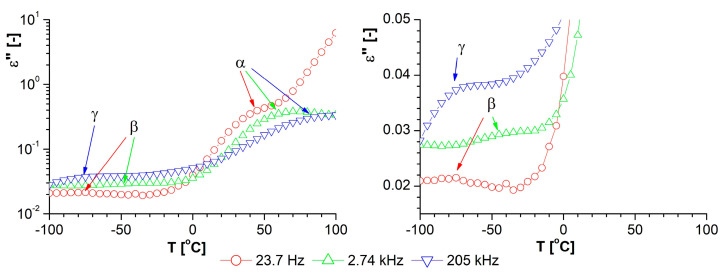
The imaginary component of the complex dielectric permittivity of the plain epoxy network LCEN in a wide temperature range, at selected frequencies. The plot on the right side enlarges the low-intensity region to emphasize the occurring relaxations. A corresponding plot for the real component of the complex dielectric permittivity can be found in the Supplementary Data (Figure S5).

**Figure 7:**
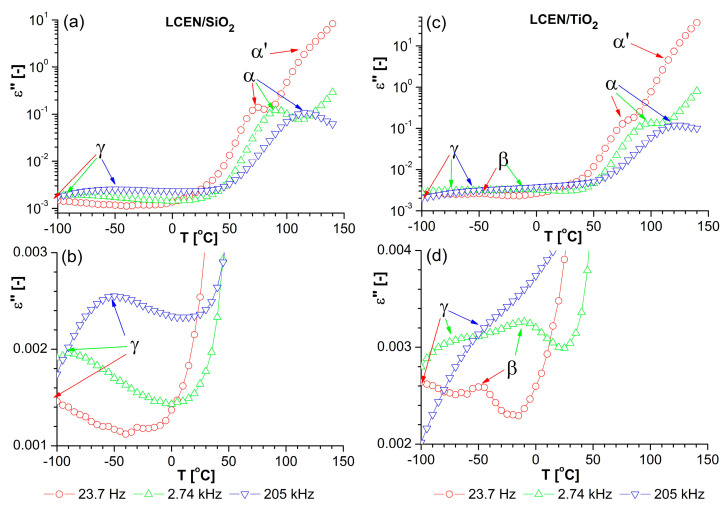
The imaginary component of the complex dielectric permittivity of the cured epoxy network modified with SiO_2_ (**a**,**b**) or TiO_2_ (**c**,**d**) in a wide temperature range, at selected frequencies. The graphs (**b**,**d**) show a different y-axis scale, allowing better observation of the secondary relaxation processes.

To support a more detailed analysis, the imaginary part of the complex permittivity is also presented as a 2D plot in a wide range of temperatures, at three selected frequencies (Figure 6 and Figure 7). The presence of the three relaxation processes is clearly visible in this representation (Figure 6—the plot on the right side is a scaled view in the low-intensity region). The α-process appearing in all cured products (Figure 6 and Figure 7) is related to the vitrification phenomenon. The secondary β-process is dominant in temperatures below *T_g_*. It is usually associated with the motion of polar groups in the mesogen. This relaxation is not visible in the matrix with SiO_2_, as it is most probably masked by the strong γ-relaxation.

An additional γ-relaxation appears at even lower temperatures in the pure matrix (LCEN—Figure 6) and in the matrices with SiO_2_ or TiO_2_ (Figure 7). The presence of this process could be linked to conformational changes in the free part of the carbon chain. In both composites, the α’-process is also visible at high temperatures (Figure 7). This additional process was not observed in the monomer or in the pure matrix; therefore, it is naturally attributable to the presence of fillers. This is a so-called Maxwell–Wagner relaxation, which also appears in other heterogenic composites. In our case, this relaxation does not influence the dielectric response at lower temperatures.

### 3.3. Properties of the Obtained Organic/Inorganic Matrices in Dielectric Spectroscopy

**Figure 8:**
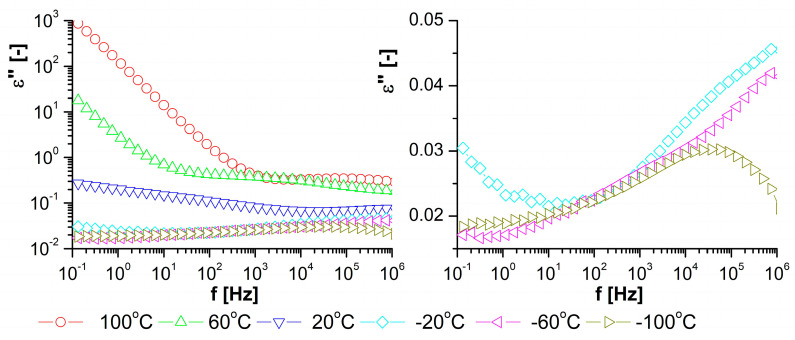
Examples of the relaxation processes in the LCEN matrix, observed in the imaginary part of the complex dielectric permittivity vs. frequency representation at a few selected temperatures. The plot on the right side enlarges the low-intensity region to emphasize the occurring relaxations. A corresponding plot for the real component of the complex dielectric permittivity can be found in the Supplementary Data (Figure S6).

**Figure 9:**
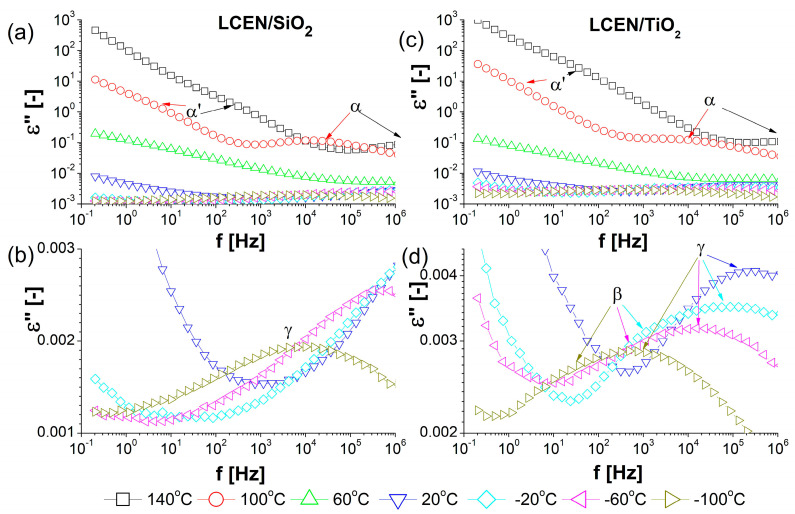
Examples of the relaxation processes observed in the frequency representation of the imaginary component of the complex dielectric permittivity at a few selected temperatures in LCEN/SiO_2_ (**a**,**b**) and LCEN/TiO_2_ (**c**,**d**). Plots (**a**,**c**) show the overall dielectric response; plots (**b**,**d**) enlarge the low-intensity regions to emphasize the occurring relaxations.

The obtained products exhibit differences in their dielectric responses, which can lead to changes in the physical properties of those products. For example, changes in the glass transition temperature (*T_g_*) translate to different mechanical properties at room temperature. To study the processes appearing in the investigated materials in detail, we plotted changes in the imaginary part of the complex dielectric permittivity against frequency for a few selected temperatures. Some examples of relaxation processes visible in the frequency representation are shown in Figure 8 and Figure 9. Similarly to the temperature representation, three processes—α, γ and α’ (at high temperatures)—are visible in both composites, and an additional β-process appears in the LCEN/TiO_2_ composite at low temperatures. As can be seen in Figure 9d, processes β and γ in LCEN/TiO_2_ overlap, giving a very broad double peak. Also, in LCEN/SiO_2_ the loss peak is very broad, which may indicate that it may consist of two overlapping processes and the β-process is hidden by the more intensive γ-process. However, it is impossible to separate them reliably.

**Figure 10:**
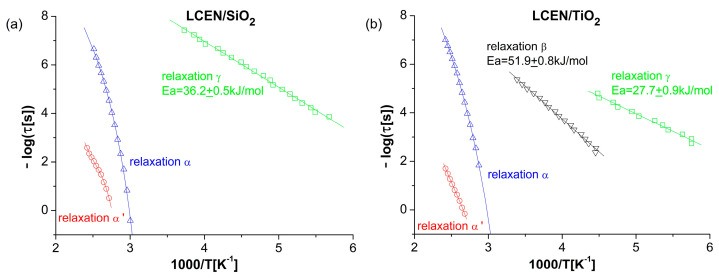
The temperature dependency of the relaxation time for all processes observed in LCEN/SiO_2_ (**a**) and LCEN/TiO_2_ (**b**) materials: α-process with a characteristic VFT curve and β- and γ-processes with an Arrhenius-type dependency.

For all the observed relaxations processes, the Havriliak–Negami formula with the added DC conductivity contribution (Equation (1)) was fitted to the experimental data. Figure 10 presents plots of the relaxation time vs. temperature for all the processes detected in the investigated composites. The relaxation times follow the Arrhenius dependency (activation energies of these relaxation are given in Figure 10) for the β- and γ-processes, and the VFT relation for the α-process. The VFT model lets us estimate *T_g_* and other parameters. The values obtained for the investigated composites are gathered in Table 4 against values of these parameters for the pure matrix. It is worth noting that the glass transition temperatures determined from both DSC and BDS measurements are very close to each other. In both cases, the addition of the filler to the pure epoxy matrix causes the glass transition temperature to increase above room temperature. Furthermore, the fragility index increases significantly in matrices enriched with fillers. As a result, it can be concluded that stable thermosets with lower brittleness in room conditions were created.

An additional β-process occurring in the LCEN/TiO_2_ matrix points toward some possibility of chain movement, which would be a beneficial factor contributing to the greater flexibility of the product also at low temperatures. To summarize both composites, the epoxy resin modified with titanium dioxide requires a slightly lower crosslinking temperature (or shorter time) and allows for some mobility in the chains at lower temperatures.

## 4. Conclusions

In this work, we created thermosets based on a nematic monomer (NTEM) whose molecular structure includes epoxy groups attached to long aliphatic chains in nonterminal locations. The monomer was cured with the diamine DDM and modified with titanium dioxide or silicon dioxide. The appropriate curing conditions for each material were established by studying the curing process and its products. Crosslinking conditions were determined in detail for each mixture based on calorimetric measurements, as well as on optical and electrical observations conducted during the reaction. In each mixture, the components have slightly different melting points and different reaction temperatures; hence, the selection of crosslinking conditions must consider all these factors and be made separately for each mixture. Based on this research, we can conclude that the matrix modified with titanium dioxide needs a slightly lower curing temperature (or shorter time) for isothermal curing than the one with silicon dioxide.

The composites showed some differences, not only in the temperatures of glass transition (*T_g_*) but also in the molecular dynamics. These differences may influence the mechanical properties (elasticity) of the polymer networks. Dielectric observations revealed the presence of the α-process and second relaxation in both examined epoxy composites, and an additional third relaxation appearing only in the NTEM composite modified with titanium dioxide. The glass transition temperatures determined from both DSC and BDS measurements were very close to each other.

To sum up, it should be stated that the introduction of additional fillers into the plain matrix modified certain thermal properties and the dielectric response. Firstly, in matrices with fillers, the glass transition temperature was shifted up by more than 30 °C in comparison with the pure matrix. Bearing in mind that slight changes in the glass transition temperature may result from the crosslinking conditions, cooling rate and thermal history of the sample, such a significant increase (above 30 °C) clearly proves the influence of the modifiers used. Additionally, the glass transition temperatures obtained from different measurements (DSC and BDS) of products hardened in the same conditions are close to each other. Apart from the glass transition temperature, significant differences in the modified matrices are also visible in other parameters obtained from the BDS experiment (including a visible increase in the fragility index).

In conclusion, we obtained stable organic/inorganic composites based on a liquid crystal epoxy matrix and exhibiting elevated *T_g_* temperatures, which makes the final product well suited for use at room conditions.

## Figures and Tables

**Table 1:** Temperatures of phase transitions in the NTEM monomer [35].

Phase Transition	Temperature [°C]	Stage
Solid (S) to Nematic (N) Nematic (N) to Isotropic liquid (I)	117 142	heating
Isotropic liquid (I) to Nematic (N) Nematic (N) to Solid (S)	140 113	cooling

**Table 2:** Composition of the studied mixtures and their assigned acronyms.

Mixture	Acronym	Percentage of Oxides in the Mixture
NTEM/DDM	LCEN	0
NTEM/DDM + SiO_2_	LCEN/SiO_2_	8.6%
NTEM/DDM + TiO_2_	LCEN/TiO_2_	9.7%

**Table 3:** Curing conditions for the mixtures: temperature and time.

Sample Acronym	Curing Temperature [°C]	Curing Time [h]
LCEN	185	3
LCEN/SiO_2_	195	3
LCEN/TiO_2_	190	2

**Table 4:** Glass transition temperatures of the investigated composites, obtained with different techniques (BDS and DSC), and derived parameters. Parameters of the pure epoxy matrix are included for comparison.

Sample	*T_g_* [K] from DSC	*T_g_* [K] from BDS	*T_v_* [K]	*D*	*m*	*E*_a_′ (*T_g_*) [kJ mol^−1^]
LCEN	283 [35]	289 ± 10	182 ± 9	23.9 ± 4.5	47.7	264
LCEN/TiO_2_	322.7	323 ± 8	239 ± 7	14.6 ± 1.9	69.4	429
LCEN/SiO_2_	319.1	326 ± 5	271 ± 4	7.1 ± 0.8	90.1	562

## Data Availability

The original contributions presented in the study are included in the article/Supplementary Material, further inquiries can be directed to the corresponding author.

## Supplementary Materials

The following supporting information can be downloaded at: https://www.mdpi.com/article/10.3390/polym16101320/s1, Figure S1. Examples of original results showing the imaginary component of complex dielectric permittivity along with measurement errors that depend on the temperature and frequency at which the measurement was made: (a) LCEN/SiO_2_, (b) LCEN/TiO_2_; Figure S2. The examples of deconvolution of the imaginary component of complex dielectric permittivity (taken directly from WinFit software) for LCEN/SiO_2_ for chosen temperatures: (a) 110 °C, (b) 100 °C, (c) −90 °C, (d) −100 °C; Figure S3. The examples of deconvolution of the imaginary component of complex dielectric permittivity (taken directly from WinFit software) for LCEN/TiO_2_ for chosen temperatures: (a) 140 °C, (b) 100 °C, (c) −80 °C, (d) −90 °C; Figure S4. DSC thermograms for mixtures based on the investigated monomer cured with DDM and with SiO_2_ or TiO_2_ fillers - the cooling route occurred directly after curing process shown in Figure 1 (in main text); Figure S5. Examples of the real component of the complex dielectric permittivity at a few selected frequencies in LCEN; Figure S6. Examples of the real component of the complex dielectric permittivity at a few selected temperatures in LCEN. The plot on the right side enlarges the low-intensity region to emphasize the occurring relaxations.
