# Supplementary material for: Modification of the Dielectric and Thermal Properties of Organic Frameworks Based on Nonterminal Epoxy Liquid Crystal with Silicon Dioxide and Titanium Dioxide
Source: Polymers (Basel). 2024 May 8;16(10):1320. doi: 10.3390/polym16101320 (PMC11125083; doi:10.3390/polym16101320)
Supplement: Supplementary file 1 [file polymers-16-01320-s001.zip › polymers-2918909-supplementary.pdf]

*Suplementary Data*

# Modification of the Dielectric and Thermal Properties of Organic Frameworks Based on Nonterminal Epoxy Liquid Crystal with Silicon Dioxide and Titanium Dioxide


**Lidia Okrasa [1,*], Magdalena Włodarska [2], Maciej Kisiel [3] and Beata Mossety-Leszczak [3]**

[1] Department of Molecular Physics, Lodz University of Technology, Żeromskiego 116, 90-924 Lodz, Poland

[2] Institute of Physics, Lodz University of Technology, Wólczańska 217/221, 93-005 Lodz, Poland; magdalena.wlodarska@p.lodz.pl

[3] Department of Industrial and Materials Chemistry, Rzeszow University of Technology, al. Powstańców Warszawy 12, 35-959 Rzeszow, Poland; m.kisiel@prz.edu.pl (M.K.); mossety@prz.edu.pl (B.M.-L.)

* Correspondence: lidia.okrasa@p.lodz.pl


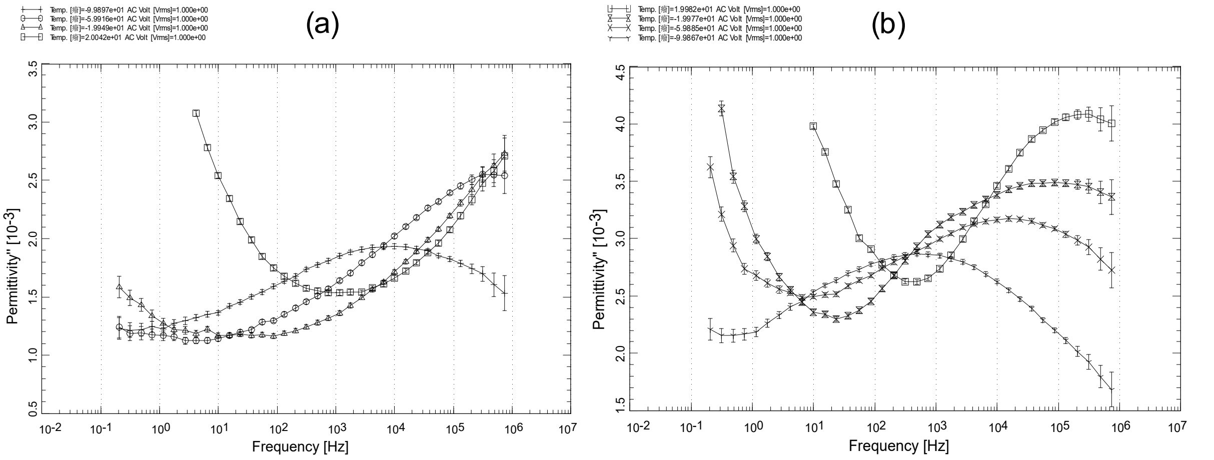


**Figure S1.** Examples of original results showing the imaginary component of complex dielectric permittivity along with measurement errors that depend on the temperature and frequency at which the measurement was made: (a) LCEN/$SiO_2$, (b) LCEN/$TiO_2$.

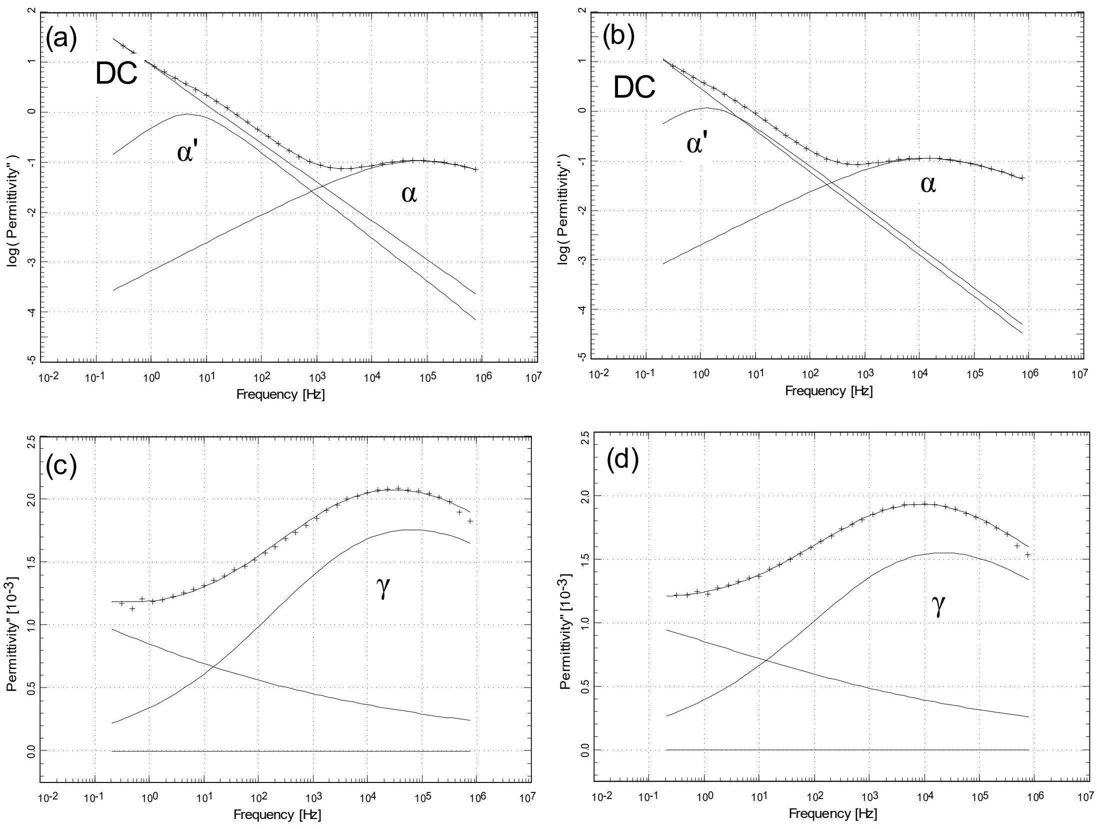


**Figure S2.** The examples of deconvolution of the imaginary component of complex dielectric permittivity (taken directly from WinFit software) for LCEN/$SiO_2$ for chosen temperatures: (a) 110°C, (b) 100°C, (c) -90°C, (d) -100°C.

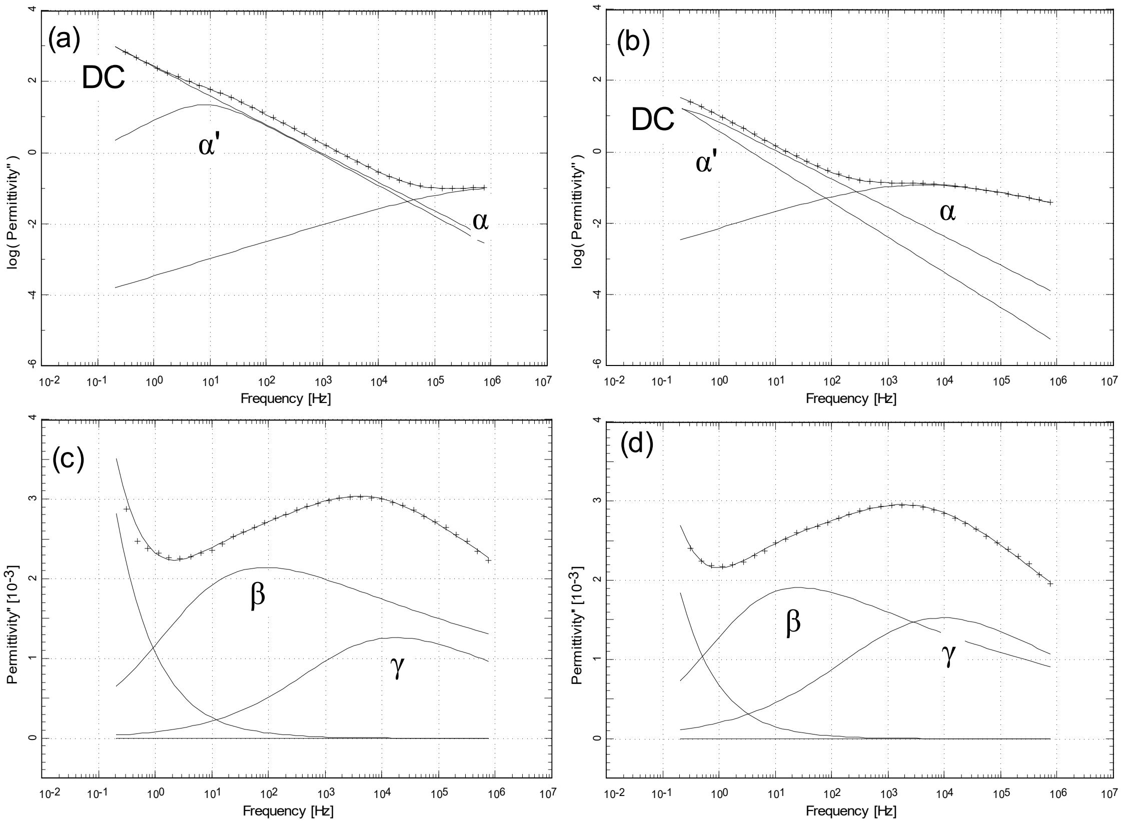


**Figure S3.** The examples of deconvolution of the imaginary component of complex dielectric permittivity (taken directly from WinFit software) for LCEN/$TiO_2$ for chosen temperatures: (a) 140°C, (b) 100°C, (c) -80°C, (d) -90°C.

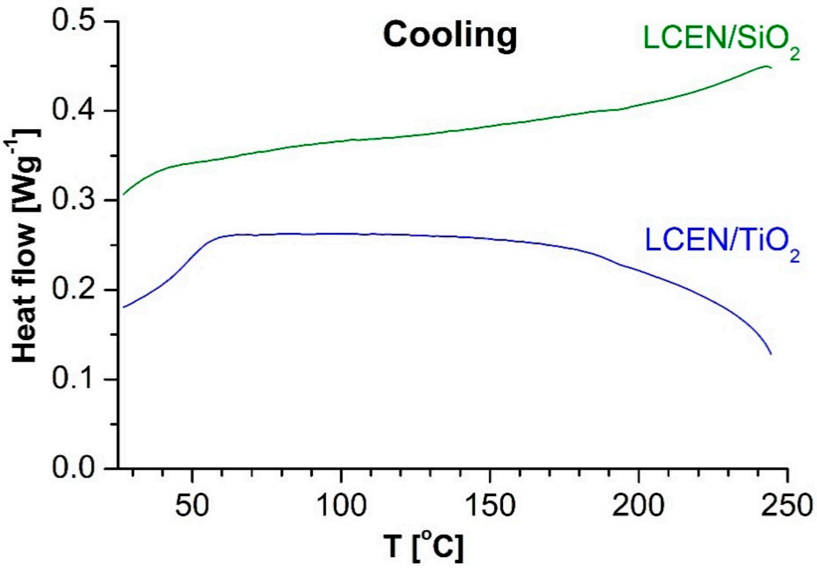


**Figure S4.** DSC thermograms for mixtures based on the investigated monomer cured with DDM and with $SiO_2$ or $TiO_2$ fillers - the cooling route occurred directly after curing process shown in Figure 1 (in main text).

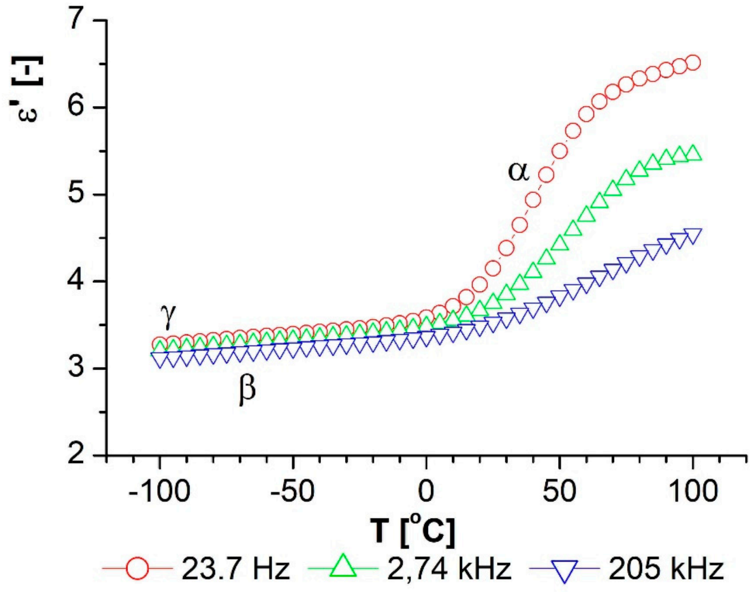


**Figure S5.** Examples of the real component of the complex dielectric permittivity at a few selected frequencies in LCEN.

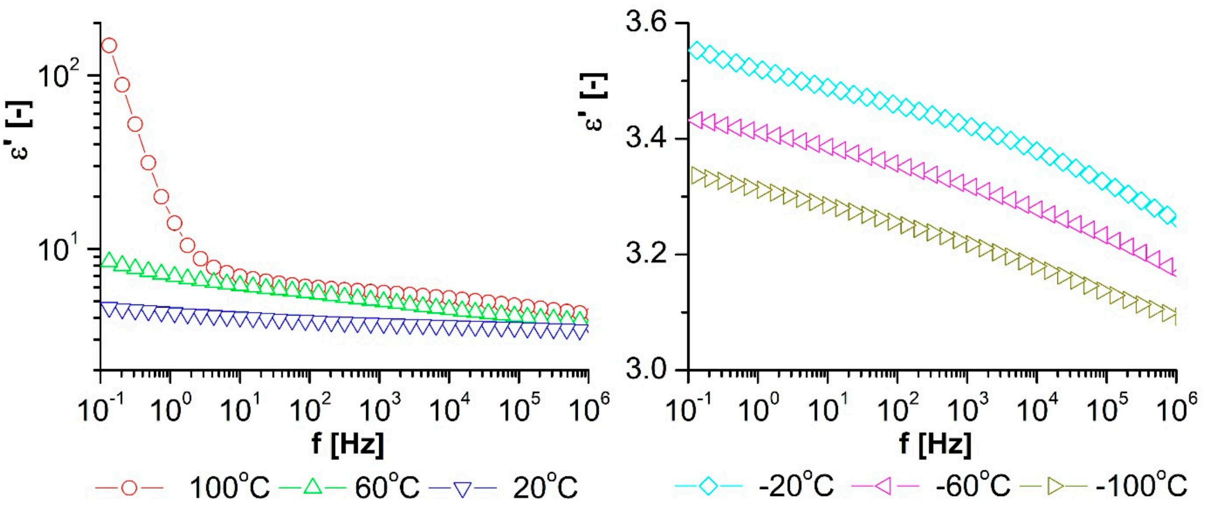


**Figure S6.** Examples of the real component of the complex dielectric permittivity at a few selected temperatures in LCEN. The plot on the right side enlarges the low-intensity region to emphasize the occurring relaxations.
